# Preventative Effect of an Herbal Preparation (HemoHIM) on Development of Airway Inflammation in Mice via Modulation of Th1/2 Cells Differentiation

**DOI:** 10.1371/journal.pone.0068552

**Published:** 2013-07-02

**Authors:** Jong-Jin Kim, Hyun Wook Cho, Hae-Ran Park, Uhee Jung, Sung-Kee Jo, Sung-Tae Yee

**Affiliations:** 1 Department of Biology, Sunchon National University, Suncheon, Republic of Korea; 2 Radiation Research Division for Bio-Technology, Advanced Radiation Technology Institute, Jeongeup Campus of Korea Atomic Energy Research Institute (KAERI), Jeongeup, Republic of Korea; 3 Department of Pharmacy, Sunchon National University, Suncheon, Republic of Korea; Institute for Virus Research, Laboratory of Infection and Prevention, Japan

## Abstract

HemoHIM, an herbal preparation of three edible herbs (Angelica gigas Nakai, Cnidium officinale Makino, Paeonia japonica Miyabe) is known to increase the Th1 immune response as well as reduce the allergic response in human mast cells. Here, our goal was to determine whether or not HemoHIM could induce Th1 cell differentiation as well as inhibit the development of airway inflammation. To study Th1/Th2 cell differentiation, naive CD4^+^ T cells isolated from C57BL/6 mouse spleens were cultured with or without HemoHIM. To examine airway inflammation, C57BL/6 mice were fed HemoHIM for 4 weeks before sensitization and provocation with ovalbumin (OVA). In an *in vitro* experiment, naive CD4^+^ T cells displayed increased Th1 (IFN-γ^+^ cell) as well as decreased Th2 (IL-4^+^ cell) differentiation in a HemoHIM concentration-dependent manner. Furthermore, in an airway inflammation mice model, eosinophil numbers in BALF, serum levels of OVA-specific IgE and IgG1, and cytokine (IL-4, IL-5, and IL-13) levels in BALF and the supernatant of splenocytes all decreased upon HemoHIM (100 mg/kg body weight) pretreatment (4 weeks). These results show that HemoHIM attenuated allergic airway inflammation in the mouse model through regulation of the Th1/Th2 balance.

## Introduction

Airway inflammation is an important symptom of asthma. The prevalence of asthma has increased considerably in recent decades, making it one of the most common chronic disorders worldwide [1]. Accordingly, primary prevention strategies to combat asthma are urgently needed, but they must be based on a sound understanding of the various determinants of the onset of asthma [2].

Naive CD4^+^ T cells are activated by antigen-presenting cells (APCs) to differentiate into one of at least two distinct T helper cell subsets, type 1 helper (Th1) cells or type 2 helper (Th2) cells. Usually, allergic diseases are caused by exaggerated Th2-type immune responses such as innocuous environmental antigens [3]–[8]. Bronchial asthma is characterized by airway hyperresponsiveness, eosinophilic airway inflammation, and increased immunoglobulin E (IgE) levels [9]–[11]. In particular, eotaxin, RANTES, IL-4, IL-5, and IL-13, which are produced by Th2 cells, are all related to airway hyperresponsiveness as well as inflammatory changes through activation of eosinophils and IgE production by B cells [12]–[14]. Since the influx and differentiation of Th2 cells are important factors in the development and aggravation of asthma, recent studies have targeted the activation of Th2 cells or regulation of the Th1/2 balance to prevent and treat asthma [15]–[17].

A new herbal preparation, HemoHIM, prepared by adding its polysaccharide fraction to the hot water extract of an herbal mixture consisting of Angelica Radix, Cnidii Rhizoma, and Paeonia Radix [18], was designed to protect self-renewal of tissues and promote immune system recovery against oxidative stresses such as irradiation [19]. HemoHIM was reported to inhibit various activities of human mast cells [20]. Additionally, HemoHIM is able to restore immune function in aged or gamma-irradiated mice based on increased growth and secretion of cytokines (IL-2, IL-12, and IFN-γ) in spleen cells, increased IFN-γ, and decreased IL-4 in lymphocytes [21], [22] **.** Further, HemoHIM has been shown to have anti-tumor effects during radiotherapy and chemotherapy [23], [24].

Recently, various asthma studies have been performed [25], [26]. In this study, we evaluated the preventative effect of HemoHIM on ovalbumin (OVA)-induced airway inflammation in mice.

## Materials and Methods

### Animals and Ethics Statement

Seven- to 8-week-old female C57BL/6 mice were bred and maintained under specific pathogen-free conditions at DAE HAN Biolink (Eumseong, Korea). Animals were housed at a controlled temperature of 22±2°C and at 50±5% relative humidity. Mice were housed in polycarbonate cages and fed a standard animal diet with water. All mice were treated in strict accordance with Sunchon National University Institutional Animal Care and Use Committee (SCNU IACUC) guidelines for the care and use of laboratory animals. All procedures were approved by the SCNU IACUC. All experiments were performed under sodium pentobarbital anesthesia, and all efforts were made to minimize suffering.

### Preparation of HemoHIM

Equal amounts of three edible medicinal herbs −Angelica Radix (root of Angelica gigas Nakai), Cnidii Rhizoma (rhizome of Cnidium officinale Makino), and Paeonia Radix (root of Paeonia japonica Miyabe) − were mixed and decocted for 4 h in boiling water to obtain total extract (HIM-I). HIM-I was divided into two parts. The ethanol-insoluble polysaccharide fraction was obtained from one part of HIM-I by precipitation in 80% (vol/vol) ethanol. This polysaccharide fraction was then added to the other part of HIM-I to obtain HemoHIM [18]–[24], which was freeze-dried and kept at −20°C. HemoHIM was composed of carbohydrates (60.4%), protein (6.0%), and other components (33.6%). Validation of HemoHIM was performed by high-performance liquid chromatography analysis of three indicator phytochemicals of each ingredient herb: nodakenin (0.58±0.04%) for Angelica Radix, chlorogenic acid (0.33±0.05%) for Cnidii Rhizoma, and paeoniflorin (1.32±0.15%) for Paeonia Radix.

### Cells

Naive CD4^+^ T cells were isolated from C57BL/6 spleens by using a CD4^+^CD62L^+^ T Cell Isolation Kit II and Separation Columns (MACS, Auburn, USA) according to the manufacturer’s instructions. T cell-depleted spleen APCs (TDS) were obtained from the spleens. Briefly, spleen cells were incubated for 60 min with anti-Thy1.2, followed by the addition of rabbit complement, washing with medium, and treatment with mytomycin C (Sigma; Louis, USA).

### In Vitro Priming of Naive CD4^+^ T cells

Priming of naive CD4^+^ T cells (1×10^5^ cells/2 mL/culture) was conducted using 1 µg/mL of anti-CD3 (145-2C11) together with MMC-treated T cell-depleted splenic APCs (TDS) (3×10^6^ cells/2 mL/culture) in a 24-well plate (SPL; Pocheon-Si, Korea). In some cases, soluble anti-CD3 and TDS were replaced by immobilized (2 µg/mL) anti-CD3 and anti-CD28 (37.51). Cultures received only medium or 1–100 µg/mL of HemoHIM. Other cultures were supplemented with 5 µg/mL of anti-IL-4 (11B11) plus 1,000 U/mL of rmIL-12 (BD Biosciences; San Diego, USA) or 5 µg/mL of anti-IL-12 (C17.8) plus 1,000 U/mL of rmIL-4 (R&D System). CD4^+^ T cells primed for 4 days were expanded into four wells with fresh medium containing 2.5 ng/mL of rhIL-2 (BD Biosciences; San Diego, USA) and cultured for another 2 days. These primed T cells were then re-stimulated at 1×10^6^ cells/mL with 50 µg/mL of PMA and 1 µM ionomycin for 5 h in the presence of 5 µg/mL of brefeldin A for the last 3 h, after which they were assayed for intracellular cytokines by FACS. Intracellular transcription factor was measured in naive CD4^+^ T cells 24 h after stimulation by anti-CD3 and anti-CD28.

### Intracellular Staining

CD4^+^ T cells were stained with biotin-anti-CD4 in the presence of anti-FcR (2.4G2), followed by incubation with streptavidin-CyChrome. Cells were then fixed with 4% paraformaldehyde in PBS and permealized with 0.1% saponin, followed by staining with FITC-anti-IFN-γ, PE-anti-IL-4, Alexa Fluor® 647 mouse anti-T-bet, and PE mouse anti-GATA-3 (BD Biosciences; San Diego, USA). CD4^+^ T cells were gated and analyzed on a FACScanto II (BD Biosciences).

### Sensitization and Provocation of Airway Inflammation with Ovalbumin

**Figure 1:**
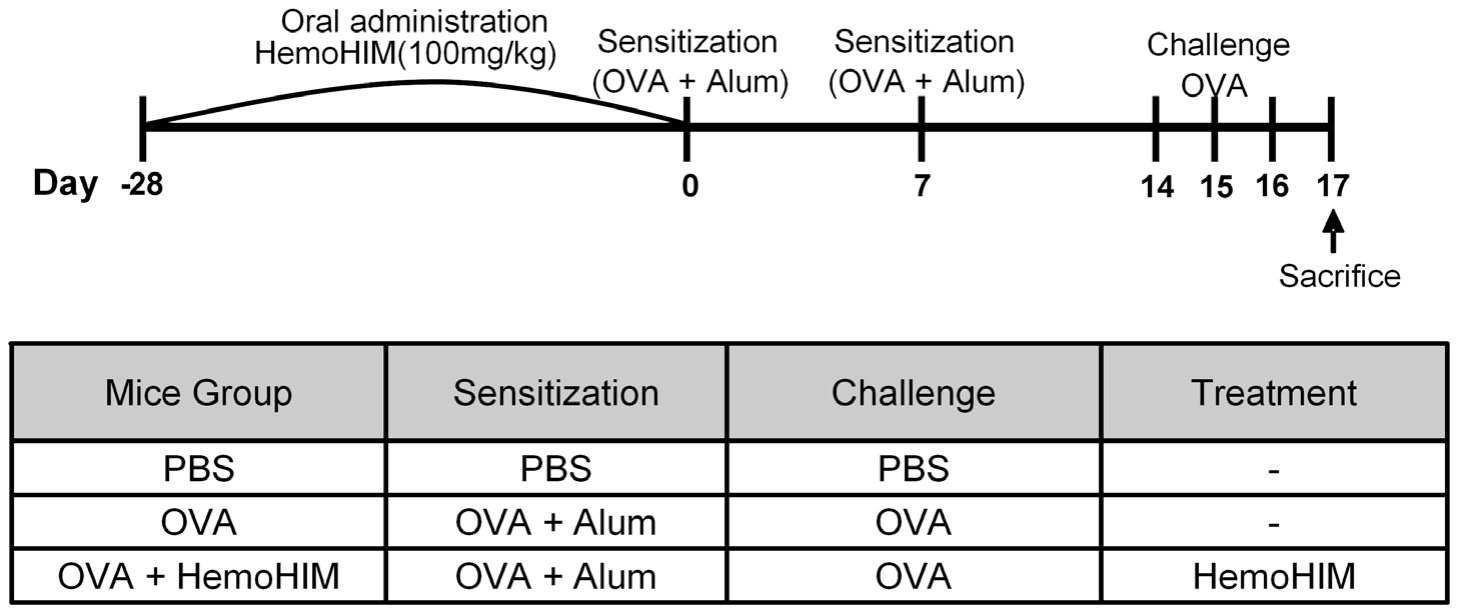
Experimental protocol for induction of airway inflammation along with treatment scheme.

HemoHIM was orally administered at 100 mg/kg in PBS (200 µL/mice). The pretreatment group was treated for 4 weeks before OVA sensitization (Fig. 1). C57BL/6 mice were sensitized by intraperitoneal injection of 10 µg of OVA and 1 mg of Imject Alum (Pierce) in 0.2 mL of saline on days 0 and 7. Control mice were also injected with an equal volume of PBS. On days 14–16, mice received 10 µg of OVA dissolved in 50 µL of saline intranasally under anesthesia with pentobarbital. On day 17, bronchoalveolar lavage (BAL) of lethally anesthetized mice was performed with four 0.5 mL aliquots of saline. After centrifugation (1200 rpm, 4°C, 5 min), supernatant of BAL fluid (BALF) recovered from 2 mL of instilled saline was stored at −20°C until assayed for cytokines by ELISA. Red blood cells in BALF were lysed with tris-buffered ammonium chloride, and nucleated cells were counted using a hemacytometer. Differential cell counts were obtained from cytospin preparations of BAL cells after Diff-Quik (Sysmex; Kobe, Japan) staining. Collected sera were stored for -20°C until assayed for immunoglobulin by ELISA.

### Histological Examination of Mouse Lung Tissue

After BAL, the mouse left lungs were harvested and paraffin embedded after fixation in formalin. Four-micron sections were cut and stained with Hematoxylin & Eosin (H&E) and PAS. For immunohistochemistry, paraffin-embedded sections were deparaffinized, and antigen retrieval was performed in 10 mM sodium citrate buffer, pH 7.4, for 5 minutes (three times) at 110°C. Slides were washed at room temperature and hydrated in PBS for 10 minutes. Endogenous peroxidase activity was then quenched with 3% hydrogen peroxidase for 10–20 minutes at room temperature. Sections were then blocked with 10% normal goat serum in PBS containing 1% BSA and 0.1% Tween-20 for 1 hour. Endogenous avidin and biotin were blocked, following the manufacturer’s instructions. Samples were then stained with antibody against caspase-1 (1∶200; Abcam). Incubations were done overnight at 4°C. Biotinylated secondary antibodies were used at 2 µg/ml and were detected with horseradish peroxidase, using the Vectastain Elite ABC (Vector Laboratories) as per manufacturer’s instructions.

### Stimulation of Spleen Cells

Spleen cells obtained from mice 24 h after the last challenge were cultured at 1×10^7^ cells/mL with 600 µg/mL of OVA. Culture supernatants collected after 24, 48, and 72 h of culture were assayed for cytokines by ELISA.

### Cytokine and Immunoglobulin ELISA

Levels of IL-2, IL-4, IL-5, IL-13, and IFN-γ in culture supernatants or BALF were assayed by ELISA. The lower detection limits of these assays were 1.11 pg/mL (IL-2, IL-4, and IL-5) and 10 pg/mL (IL-13 and IFN-γ). OVA-specific IgG1, IgG2a, and IgE concentrations in serum were assayed using OVA as the capture Ag and biotinylated anti-mouse IgG1, IgG2a, or IgE mAb (BD Biosciences; San Diego, USA) as the detection Ab. Total serum IgE levels were determined by ELISA using paired mAbs specific for standard mouse IgE and IgE (BD Biosciences; San Diego, USA).

### Statistical Analysis

All data are presented as the means ± standard deviation, and data were evaluated by one-way ANOVA using SPSS (SPSS Inc., Chicago). Differences between the means were assessed using Duncan’s multiple-range test. Statistical significance was considered at p<0.01 and p<0.05.

## Results

### Modulation of Naive CD4^+^ T Cell Differentiation

**Figure 2:**
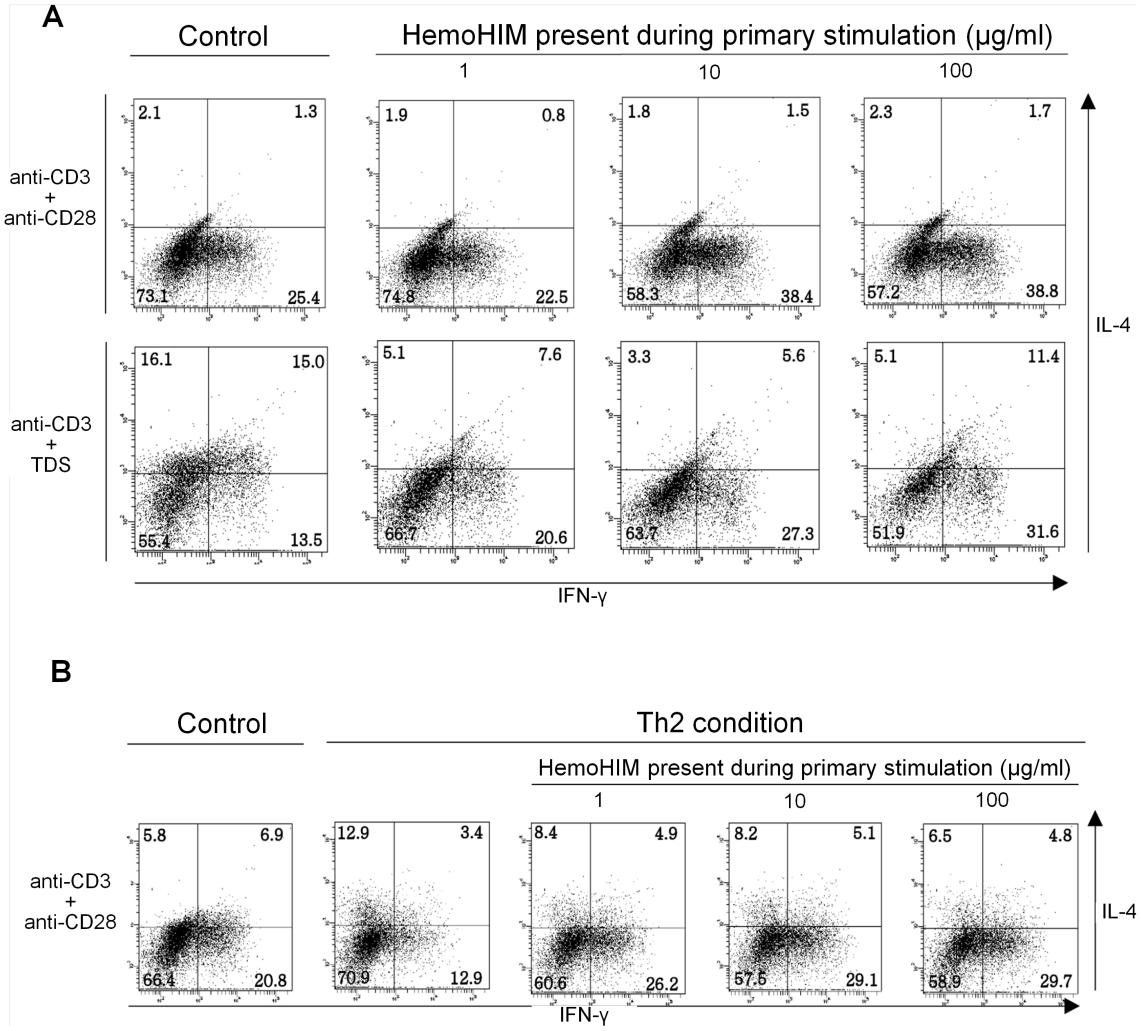
Cytokine production profiles of T cells in the presence of HemoHIM. (A) Flow cytometric analysis of intracellular IL-4 and IFN-γ staining in CD4^+^ T cells primed with anti-CD3 plus anti-CD28 or anti-CD3 plus TDS with the indicated concentration of HemoHIM. (B) CD4^+^ T cells primed with anti-CD3 plus anti-CD28 in the absence (normal conditions) or presence of anti-IL-12 plus IL-4 (Th2 conditions) with the indicated concentration of HemoHIM. Data represent one of the three independent experiments.

Priming of naive CD4^+^ T cells from C57BL/6 mice resulted in the development of IL-4-producing Th2 or IFN-γ-producing Th1 cells. Further, HemoHIM augmented Th1 and suppressed Th2 development of naive CD4^+^ T cells from C57BL/6 mice in a dose-dependent manner upon stimulation with anti-CD3 plus anti-CD28 or anti-CD3 plus TDS (Fig. 2A). A similar effect of HemoHIM was observed even when naive CD4^+^ T cells were primed under Th2-polarizing conditions (anti-IL-12 plus IL-4) (Fig. 2B). Additionally, after stimulation (anti-CD3 plus anti-CD28) for 24 h, transcription factors were assayed by intracellular staining of T-bet and GATA-3. Priming of naive CD4^+^ T cells resulted predominantly in the development of GATA-3-producing Th2 and T-bet-producing Th1 cells, respectively. Further, HemoHIM augmented T-bet and suppressed GATA-3 expression in the development of naive CD4^+^ T cells (data not shown).

### Suppression of Airway Inflammation by HemoHIM

**Figure 3:**
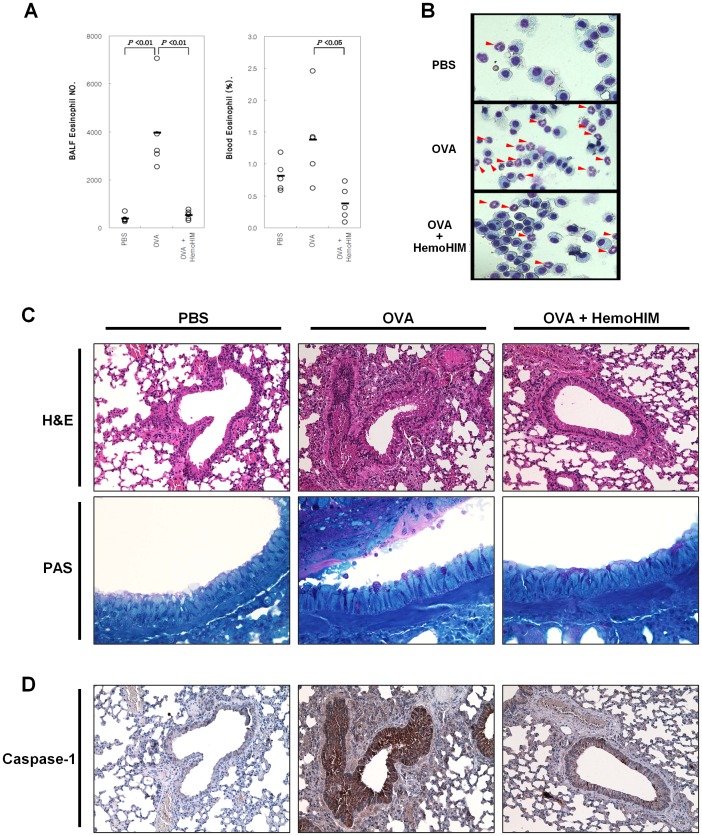
Inhibition of airway inflammation by HemoHIM. C57BL/6 mice orally administered HemoHIM (4 weeks) before being sensitized and challenged with OVA. In these mice, airway inflammation was induced as described in Materials and Methods. Blood and BALF collected 24 h after the last challenge. (A) Number of eosinophils in BALF and blood, (B) microphotographs of BALF cells stained with Diff-Quik in each group of the airway inflammation mouse model (×200). (C) Following BAL, mouse left lungs were stained with H&E (×200) and PAS (×400). (D) Caspase-1 immunohistochemistry in the lung (×200). Data represent five mice per group. ‘−’ indicate the mean of five mice. Data represent one of the three independent experiments.

In an *in vivo* test, we used an OVA-induced airway inflammation mouse model. HemoHIM-pretreated mice displayed lower total BALF cell counts compared with OVA only -sensitized mice (data not shown). Furthermore, the HemoHIM pretreatment group showed a significantly reduced eosinophil count in BALF compared with OVA only-sensitized mice (532±190 vs. 3,963±1,794, P<0.01) (Fig. 3A). Figure 3B shows microphotographs of BALF cells stained with Diff-Quik from each group of mice. H&E staining showed that peribronchial and perivascular infiltrations of lymphocytes and eosinophils were significantly heavier in the OVA group compared with the PBS group. However, infiltrations of lymphocytes and eosinophils were significantly reduced in the OVA-HemoHIM group compared with the OVA group (Fig. 3C). Further, PAS stained goblet cell number of bronchial mucosal secretions was higher in the OVA group compared with the PBS group. However, mucosal secretions decreased in the OVA-HemoHIM group compared with the OVA group (Fig. 3C). The other evidence of airway inflammation was assessed by caspase-1 immunohistochemistry. Caspase-1 expression was increased in OVA group compared with PBS group. However, caspase-1 expression was reduced in the OVA-HemoHIM group compared with the OVA group (Fig. 3D).

### Serum Levels of Total IgE and OVA-specific IgE

**Figure 4:**
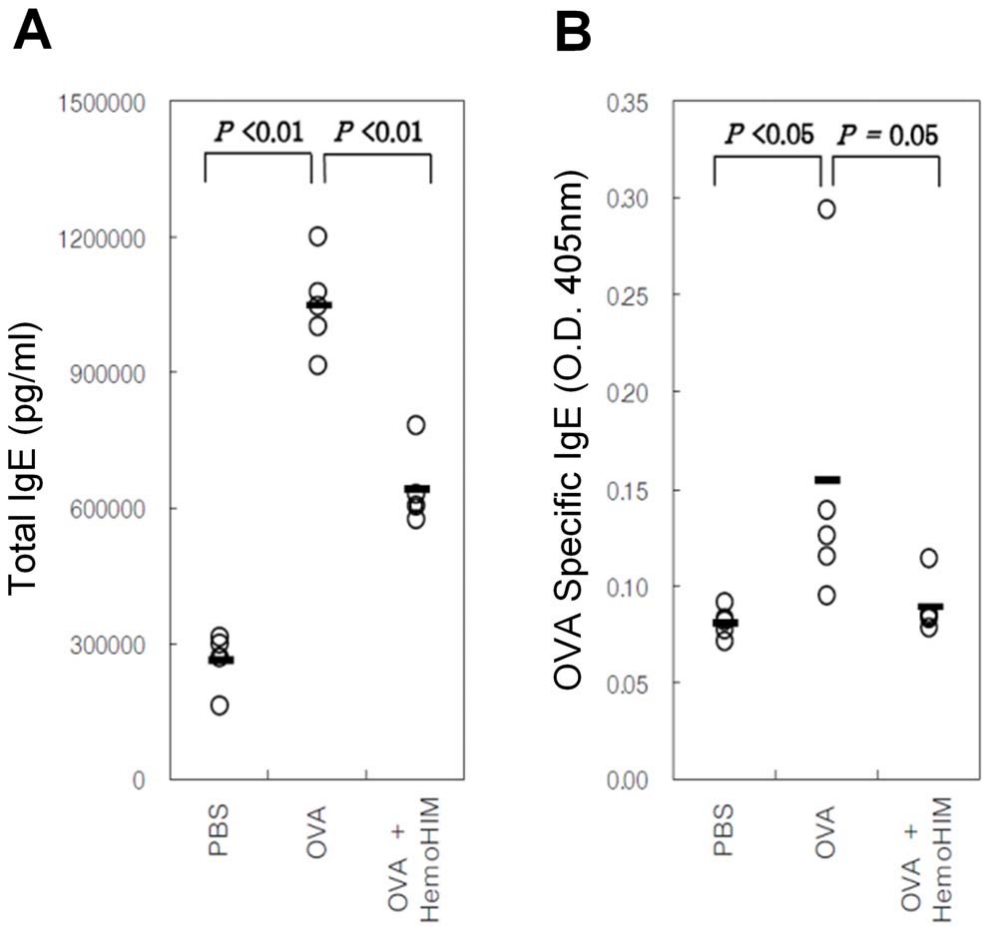
C57BL/6 mice orally administered HemoHIM (4 weeks) before being sensitized and challenged with OVA. In these mice, airway inflammation was induced as described in Materials and Methods. Blood collected 24 h after the last challenge. Serum samples were assayed for total levels of IgE and OVA-specific IgE (1/5 dilution) by ELISA. Data represent five mice per group. ‘−’ indicate the mean of five mice. Data represent one of the three independent experiments.

The total IgE level in the serum was significantly higher in the OVA group compared with the PBS group (1048±103 vs. 265±61 ng/mL, P<0.01), whereas that in the HemoHIM pretreatment group was significantly lower compared with the OVA group (640±82 vs. 1048±103 ng/mL, P<0.01; Fig. 4A). Furthermore, the OVA-specific IgE level (OD_405nm_) in the serum was significantly higher in the OVA group compared with the PBS group (0.154±0.080 vs. 0.081±0.007, P<0.05), whereas that in the HemoHIM pretreatment group was significantly lower compared with the OVA group (0.089±0.014 vs. 0.154±0.080, P = 0.05; Fig. 4B).

### Serum Levels of OVA-specific IgG1 and IgG2a

**Figure 5:**
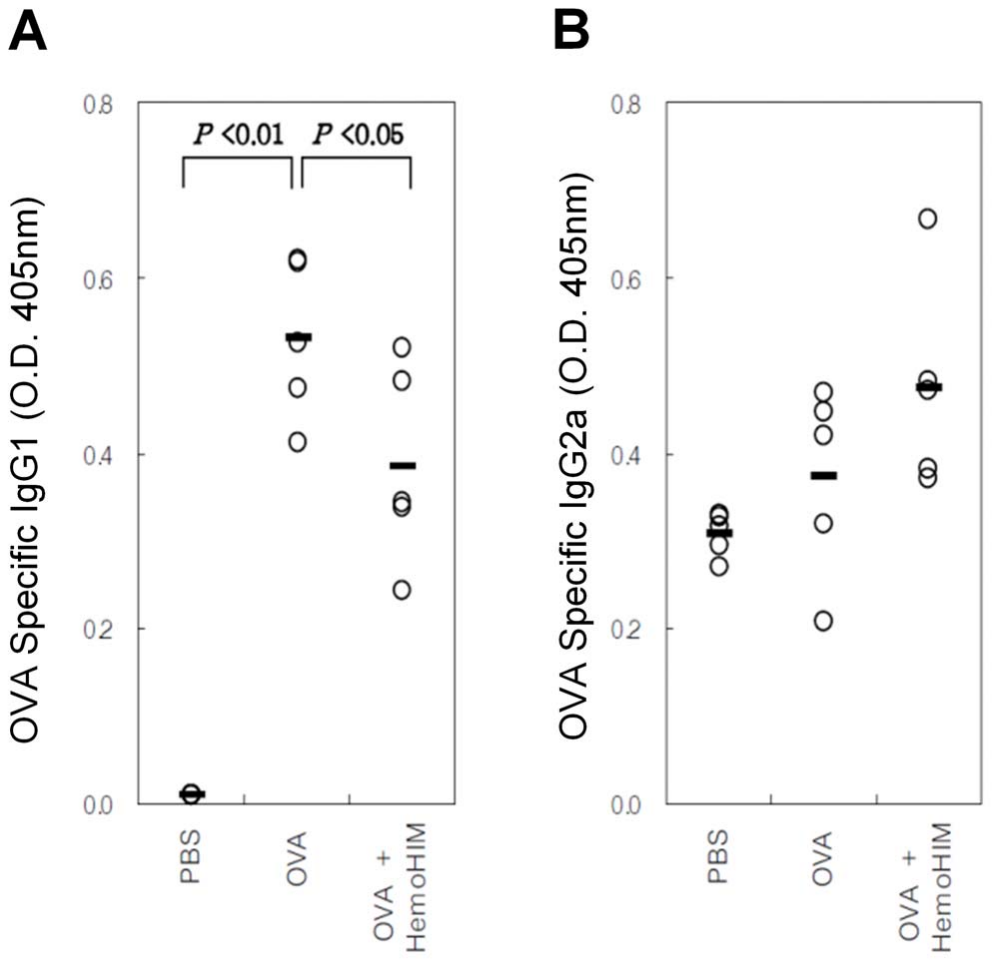
C57BL/6 mice orally administered HemoHIM (4 weeks) before being sensitized and challenged with OVA. In these mice, airway inflammation was induced as described in Materials and Methods. Blood collected 24 h after the last challenge. Serum samples were assayed for levels of OVA-specific IgG1 (1/10000 dilution) and IgG2a (1/30 dilution) by ELISA. Data represent five mice per group. ‘−’ indicate the mean of five mice. Data represent one of the three independent experiments.

The OVA-specific IgG1 level (OD_405nm_) in the serum was significantly higher in the OVA group compared with the PBS group (0.531±0.091 vs. 0.011±0.000, P<0.01), whereas that in the HemoHIM pretreatment group was significantly lower compared with the OVA group (0.386±0.113 vs. 0.531±0.091, P<0.05; Fig. 5A). Furthermore, mean OVA-specific IgG2a levels (OD_405nm_) were 0.309±0.026 in the PBS group, 0.373±0.108 in the OVA group, and 0.475±0.119 in the HemoHIM pretreatment group. The OVA-specific IgG2a level was higher in the HemoHIM pretreatment group compared with the OVA group, but the difference between the OVA and HemoHIM pretreatment groups was not statistically significant (Fig. 5B).

### Cytokine Concentration in BALF

**Figure 6:**
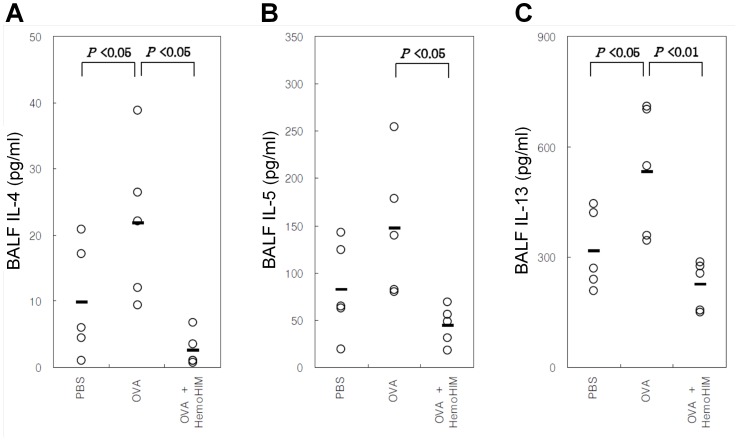
C57BL/6 mice orally administered HemoHIM (4 weeks) before being sensitized and challenged with OVA. In these mice, airway inflammation was induced as described in Materials and Methods. BLAF collected 24 h after the last challenge. Concentrations of IL-4 (A), IL-5 (B), and IL-13 (C) in BALF. Data represent five mice per group. ‘−’ indicate the mean of five mice. Data represent one of the three independent experiments.

The IL-4 concentrations (pg/mL) in BALF were 9.300±9.517 in the PBS group, 21.785±11.824 in the OVA group, and 1.493±3.760 in the HemoHIM pretreatment group. The IL-4 concentration in BALF was significantly lower in the OVA group compared with the PBS group (P<0.05), whereas that in the HemoHIM pretreatment group was significantly lower compared with the OVA group (P<0.05; Fig. 6A). Further, the concentrations of IL-5 (PBS group 82.767±50.208; OVA group 147.336±73.196; HemoHIM pretreatment group 44.7±20.0) and IL-13 (PBS group 317.3±108.5; OVA group 533.5±177.4; HemoHIM pretreatment group 225.3±66.6) in BALF were similar to the IL-4 result (Fig. 6B, C).

### Cytokine Concentration in Spleen Cell Supernatant

Spleen cells obtained from mice 24 h after the last challenge were cultured at 1×10^7^ cells/mL with 600 µg/mL of OVA. Culture supernatants collected after 72 h of culture were assayed for cytokines. The IL-5 concentration in the supernatant was significantly higher in the OVA group compared with the PBS group (P<0.05). However, the IL-5 concentration in the HemoHIM pretreatment group was lower compared with the OVA group, although the difference was not significant. The IL-13 concentration in the supernatant was significantly higher in the OVA group compared with the PBS group (P<0.01), whereas that in the HemoHIM pretreatment group was significantly lower compared with the OVA group (P<0.05). The IFN-γ concentration was different between the PBS and OVA groups, but the difference was not statistically significant. However, the IFN-γ concentration in the HemoHIM pretreatment group was significantly higher compared with the OVA group (Table 1).

**Table 1:** Decreased Th2 cytokines and increased Th1 cytokines by HemoHIM in mouse spleen cells displaying airway inflammation.

Cytokine	Concentration(pg/ml)		
	PBS	OVA	OVA+HemoHIM
IL-5	<1.11	373.83±92.09**	305.28±51.48
IL-13	82.27±5.45	406.63±105.30**	305.63±25.10**^†^**
IFN-γ	243.63±84.27	356.50±112.00	630.43±110.72††

C57BL/6 mice orally administered HemoHIM (4 weeks) before being sensitized and challenged with OVA. Spleen cells from mice were re-stimulated *in vitro* with OVA, and the culture supernatant was assayed for IL-4, IL-13, and IFN-γ by ELISA. Data are represented as the mean ± S.D. of five mice per group. Data represent one of the three independent experiments.

** *P*<0.01 compared with the PBS group.

†† *P*<0.01 or **^†^** *P*<0.05 compared with the OVA group.

## Discussion

The aim of this study was to investigate the effects of HemoHIM on the prevention of airway inflammation. Th2 cells are exacerbated in the early stages of airway inflammation through secretion of IL-4, 5, and 13. However, IFN-γ-producing Th1 cells inhibit the differentiation of Th2 cells [3], [6]. In an *in vitro* experiment, stimulation of naive CD4^+^ T cells by anti-CD3/anti-CD28 resulted in increased Th1 and decreased Th2 differentiation in a HemoHIM concentration-dependent manner. Differentiation of naive CD4^+^ T cells requires interaction with APCs. When naive CD4^+^ T cells were stimulated by anti-CD3/TDS or Th2 conditions, a similar result was observed. These data suggest that HemoHIM inhibited airway inflammation by regulating the differentiation of naive CD4^+^ T cells.

Second, we investigated the preventative effects of HemoHIM in an OVA-induced airway inflammation mouse model. Eosinophils are known as effector cells in airway inflammation due to their release of cytokines, cytotoxic granule proteins, and tissue-damaging superoxide [27]. The total number of eosinophils in BALF and the percentage of eosinophils in the blood were both significantly lower in the OVA-HemoHIM group compared with the OVA group. H&E staining revealed that peribronchial and perivascular infiltrations of inflammatory cells were reduced by HemoHIM pretreatment. Further, goblet cells are activated by Th2 cytokines such as IL-4 and IL-13 [28]. PAS staining revealed that the number of goblet cells in bronchial epithelial tissues was reduced by HemoHIM pretreatment. These results suggest that HemoHIM inhibited Th2 differentiation, thereby decreasing eosinophil counts in BALF and blood as well as activation of goblet cells. Caspase-1 known as IL-1-converting enzyme is established as the protease responsible for the processing of the key inflammatory cytokine IL-1β from an inactive precursor to an active, secreted molecule. Thus, caspase-1 is regarded as a key mediator of inflammatory processes [29]. At the immunohistochemistry, expression of caspase-1 in the lung tissue was reduced by HemoHIM pretreatment. Therefore, HemoHIM may have a preventive effect against development of airway inflammation before OVA sensitization.

Mast cells, which are activated by IgE through IgE receptor (FcεRI), secrete histamine, prostaglandin, and cytokines such as IL-3, 4, and 5 [30]. In the serum, total IgE as well as OVA-specific IgE and IgG1 levels were significantly lower in the OVA-HemoHIM group compared with the OVA group. Given these results, systemic Th2-type immune responses were reduced by treatment with HemoHIM before OVA sensitization [21]. However, OVA-specific IgG2a levels tended to increase without statistical significance in the HemoHIM-treated group. This result suggests that the Th1 immune response increased while the Th2 immune response decreased in response to HemoHIM. Th2 cytokines have been shown to induce immunoglobulin class switching of IgG1 and IgE on B cells [31] along with activation of eosinophils. The inhibition of various inflammation responses was related to reduction of Th2 cytokines in BALF, including IL-4, 5, and 13. Mice treated with HemoHIM exhibited an overall decrease in lung inflammatory responses compared with untreated mice.

As mentioned earlier, the number of Th2 cells in primary cultures treated with HemoHIM decreased, suggesting that HemoHIM inhibited the systemic Th2 immune response *in vivo*. To confirm this, the effect of HemoHIM on the systemic Th1/2 immune response was determined by examining cytokine production profiles in spleen cells. HemoHIM treatment augmented IFN-γ (Th1 cytokine) and suppressed IL-5/IL-13 (Th2 cytokine) production in spleen cells from C57BL/6 mice after re-stimulation with OVA *in vitro*. In a previous study, HemoHIM treatment in mice caused an increase in the level of IL-12, which is necessary to induce Th1 differentiation of naive CD4^+^ T cells [22]. In another report, human mast cells, which are allergic effector cells, were shown to be directly inhibited by HemoHIM [20]. These data indicate that HemoHIM suppressed the systemic Th2 immune response in mice.

In conclusion, these results suggest that oral administration of HemoHIM before sensitization can attenuate airway inflammation in mice. As current allergic airway inflammation treatments only target effector proteins or cells, these results are encouraging since HemoHIM inhibited various airway inflammation indicators by controlling Th1/Th2 differentiation during the early stage of allergic airway inflammation development.
